# Discovery of Potential Flavonoid Inhibitors Against COVID-19 3CL Proteinase Based on Virtual Screening Strategy

**DOI:** 10.3389/fmolb.2020.556481

**Published:** 2020-09-29

**Authors:** Zhongren Xu, Lixiang Yang, Xinghao Zhang, Qiling Zhang, Zhibin Yang, Yuanhao Liu, Shuang Wei, Wukun Liu

**Affiliations:** ^1^Jiangsu Collaborative Innovation Center of Chinese Medicinal Resources Industrialization, School of Medicine and Holistic Integrative Medicine, School of Pharmacy, Nanjing University of Chinese Medicine, Nanjing, China; ^2^Shenzhen Bay Laboratory, Shenzhen, China; ^3^Department of Respiratory and Critical Care Medicine, Tongji Hospital, Tongji Medical College, Huazhong University of Science and Technology, Wuhan, China

**Keywords:** COVID-19 3CL proteinase, flavonoids, rutin, virtual screening, machine learning, molecular docking

## Abstract

The outbreak of 2019 novel coronavirus (COVID-19) has caused serious threat to public health. Discovery of new anti-COVID-19 drugs is urgently needed. Fortunately, the crystal structure of COVID-19 3CL proteinase was recently resolved. The proteinase has been identified as a promising target for drug discovery in this crisis. Here, a dataset including 2030 natural compounds was screened and refined based on the machine learning and molecular docking. The performance of six machine learning (ML) methods of predicting active coronavirus inhibitors had achieved satisfactory accuracy, especially, the AUC (Area Under ROC Curve) scores with fivefold cross-validation of Logistic Regression (LR) reached up to 0.976. Comprehensive ML prediction and molecular docking results accounted for the compound Rutin, which was approved by NMPA (National Medical Products Administration), exhibited the best AUC and the most promising binding affinity compared to other compounds. Therefore, Rutin might be a promising agent in anti-COVID-19 drugs development.

## Introduction

At the end of 2019, the pneumonia of unknown cause was detected. A few weeks later a coronavirus was newly isolated, and it was first identified and regarded as the seventh member of beta coronavirus (Zhu et al., 2020). COVID-19 is an infectious disease caused by the most recently discovered coronavirus by WHO (World Health Organization). Globally, as of 9 Aug, more than 19,000,000 confirmed and 720,000 deaths were reported to WHO. Anxiously, the number of infections worldwide is still rising (Wang and Zhang, 2020). Therefore, this is a rapidly evolving emergency. It is imperative to discover and develop potential agents to treat the outbreak.

The sequence alignment demonstrated that the COVID-19 has 82% nucleotide sequence identity with human SARS-CoV (severe acute respiratory syndrome) (Chan et al., 2020; Lee and Hsueh, 2020; Xu et al., 2020). Recently, Prof. Zihe Rao’s research team successfully expressed the 3C-Like Proteinase of COVID-19. The crystal structure of COVID-19 3CLpro (PDB: 6LU7) (Liu et al., 2020) was identified, resolved in a very short time, and available in PDB (protein data bank). It is well-known that chymotrypsin-like protease is crucial in the life cycle of virus, and the protease is stable inside the coronaviruses. Thus, the COVID-19 3CLpro is a potential target for developing the new anti-COVID-19 drugs.

Since the COVID-19 3CLpro has successfully resolved, guaranteed accurate virtual screen strategies are generally considered as a rapid progress of discovering potential drugs, especially in the public health crisis. Experimental screening programs are generally considered to be time-consuming and laborious. Thus, to expedite screening possible drug molecules and prevent the outbreak, the procedure of machine learning and molecular docking were performed to narrow down the potential candidates before experimental assays (Chen et al., 2005; Berry et al., 2015). In this work, a natural compounds library including 2030 compounds (Chinese medicine compounds mostly) was chosen as the database. Some Chinese medicine compounds in the dataset are wildly used in clinic. Their mechanisms, side effects, and safety were investigated. Encouragingly, the NHC (National Health Commission of People’s Republic of China) recently issued a statement that several Chinese medicine formulas were suggested curing the patients in the early stage of infections. Of note, more and more patients were discharged from hospital after being cured by integrated treatment by combining Chinese with Western medicine in China (Cao et al., 2020; Qiu et al., 2020). In addition, it also should be mentioned that many Chinese herbal formulas such as Le-Cao-Shi, JieZe-1, and San Wu Huang Qin decoctions were used to prevent virus infection and cure the viral diseases in China (Ma et al., 2018; Zhao et al., 2019; Duan et al., 2020). The natural compounds are appropriate to be selected as a source of prototype inhibitors against COVID-19 3CLpro.

## Materials and Methods

### Molecular Descriptors and Data Sets

Chemical fingerprint recognition is a method to convert the drawn molecules into 0 and 1-bit streams. The old fingerprint type was MACCS key, which was developed by the former MDL as a fast method for substructure screening in molecular databases (Polton, 1982). Another available fingerprint is the Morgan fingerprint, a circular fingerprint (Morgan, 1965). The environment and connectivity of each atom are analyzed to a given radius, and each possibility is encoded. Therefore, Morgan fingerprint was applied to this virtual screen project as a molecular representation for machine learning. Morgan fingerprint set a molecular fingerprint by setting a radius from a specific atom to count the number of molecular structures within this radius. We can set the number of radius and bits to get different molecular fingerprints and lengths (Xue et al., 2004). Ultimately, the fingerprint length (128-bit, 256-bit, 526-bit, 1024-bit, 2048-bit) was selected in our study.

Since the binding cavity of COVID-19 3CLpro and SARS 3CLpro is extremely similar (Supplementary Figure S1), it is rational to make the learning model based on the data of inhibitors of above two proteins. The 66 active compounds and 66 inactive compounds were collected as the training and testing set data (patent: US7495011 B2) (Yamamoto et al., 2004; Chen et al., 2006; Wang et al., 2007; Jacobs et al., 2013; Adedeji and Sarafianos, 2014; García-Fernández et al., 2016; Konno et al., 2017; Hu et al., 2020; Jo et al., 2020; Yoshizawa et al., 2020). The chemical structures of compounds were drawn by ChemDraw software and translated into canonical SMILES by the RDKit python package (Shi and Borchardt, 2017). These active compounds and inactive compounds were prepared as positive samples and negative samples respectively during model training.

### Machine Learning Classifiers

Six machine learning classifiers were evaluated in this study for comparison.

#### Random Forest (Watson, 2008)

Random forest (RF) or random decision forests 9 are ensemble learning methods for classification, regression, and other tasks. The methods are operated by constructing a multitude of decision trees at training time, and they output the classes, which consist of the mode of the classes (classification) or mean prediction (regression) of the individual trees 10.

#### Support Vector Machine (Cortes and Vapnik, 1995)

The support-vector machines (SVMs, also support-vector networks) 11 are supervised learning models with associated learning algorithms, analyzing data for the classification and regression. Given a set of training examples, each marked as belonging to one or the other of two categories, the SVM training algorithm builds a model that assigns new examples to one category or the other, making a non-probabilistic binary linear classifier.

#### K-Nearest Neighbors (Altman, 1992)

The K-nearest neighbors algorithm (K-NN) 12 is a non-parametric method for the classification and regression. In both cases, the input consists of the K closest training examples in the feature space. The output depends on whether K-NN is used for the classification or regression: (1) In the K-NN classification, the output is a class membership. (2) In the K-NN regression, the output is a property value for the object. This value is the average of the values of K nearest neighbors. The K-NN is a type of instance-based learning, or lazy learning, where the function is only approximated locally and all computation is deferred until the classification.

#### Naïve Bayes 13 (Safavian and Landgrebe, 1991)

Naïve Bayes 13 is a simple technique for constructing classifiers: models that assign class labels to the problem instances, representing as vectors of feature values, where the class labels are drawn from some finite sets. There is not a single algorithm for training such classifiers, but a family of algorithms based on a common principle: all naïve Bayes classifiers assume that the value of a particular feature is independent of the value of any other feature, given the class variable.

#### Decision Tree (Tolles and Meurer, 2016)

A decision tree 14 is a flowchart-like structure in which each internal node represents a “test” on an attribute (e.g., whether a coin flip comes up heads or tails), each branch represents the outcome of the test, and each leaf node represents a class label (decision taken after computing all attributes). The paths from the root to the leaf represent classification rules.

#### Logistic Regression (Biau et al., 2008)

Logistic regression 15 is a statistical model for using a logistic function to form a model of binary dependent variable. In the regression analysis and logistic regression (or logit regression), it is always used to estimate the parameters of a logistic model (a form of binary regression).

Above all, Random Forest, Support Vector Machine, K-nearest neighbors, Naïve Bayes, Decision Tree, and Logistic Regression were realized by a machine learning package in scikit-learn (Pedregosa et al., 2011).

### Performance Measures

As for the evaluation of machine learning classifier, the AUC describes the performance of the classifier and presents the comparison between the true positive rate or sensitivity of a given model and the false positive rates (Hand, 2009). The increase in sensitivity is at the expense of the false positive rate. The AUC is a measure of the accuracy of the model. An AUC >0.5 means that the classifier could differ between the positive and negative samples effectively. A perfect classifier should be with AUC = 1.0.

### Molecular Docking

The crystal structure of COVID-19 3CLpro complex was downloaded from protein data bank (PDB ID: 6LU7) (Liu et al., 2020). A commercial database including 2030 approved natural compounds was used as the screening library (Selleck Chemicals, Houston, TX, United States).

The virtual screen procedure and refinement were conducted by Glide module in Schrödinger Maestro software (Berry et al., 2015). The 6LU7 (COVID-19 3CLpro) was performed as the acceptor and prepared in the Protein Preparation Wizard. The receptor was preprocessed, optimized, and minimized (the restrained minimization using the OPLS2005 force field). All compounds were prepared by the default settings of LigPre module. For the screening in the Glide module, the prepared receptor was imported to specify the suitable position in the Receptor Grid Generation. The grid box was generated 10 Å in X, Y, and Z direction, and the residue of His41 and Cys145 was selected as the centroid of the grid box. The Glide Ligand docking was subsequently carried out with the default settings applied (standard precision) and XP (extra precision) templates. The dataset was screened through SP docking first; the SP docking template is appropriate for screening compounds in large numbers. Subsequently, the top ligands that had been determined to be high-scoring using SP were screened by XP docking template, and the XP method is to provide a better correlation between good poses and good scores. All other settings remained default to the Ligand docking wizard.

The phase module of pharmacophore was used to generate pharmacophore hypothesis and define the pharmacophoric features using the receptor-original ligand complex 6LU7. Conformation of each hit was generated using Confgen by applying the OPLS-2005 force field (Watts et al., 2010; Rohini and Shanthi, 2018). Features were set based on the original inhibitor binding model, which contained essential binding interactions with key residues including His41, Phe140, Gln142, Cys145, His164, Glu166, Gln189, and Thr190. The phase ligand screening was subsequently carried out using the above pharmacophore hypothesis based on Glide XP scoring terms. The visualization results and the Phase Screen Score were used to examine the alignment between initial ligands and pharmacophore features.

The binding free energy of each complex was calculated using the panel of MM-GBSA technology available with Prime. This panel can also be used to calculate ligand strain energies for a set of ligands and a single receptor. The ligands and the receptor must be properly prepared beforehand, and it is common to prepare by using LigPrep and the Protein Preparation Wizard. The calculated difference between the minimized receptor-ligand complex and the minimized unbound ligand-receptor was scored by the MM-GBSA with the VGSB solution model.

## Results

**FIGURE 1:**
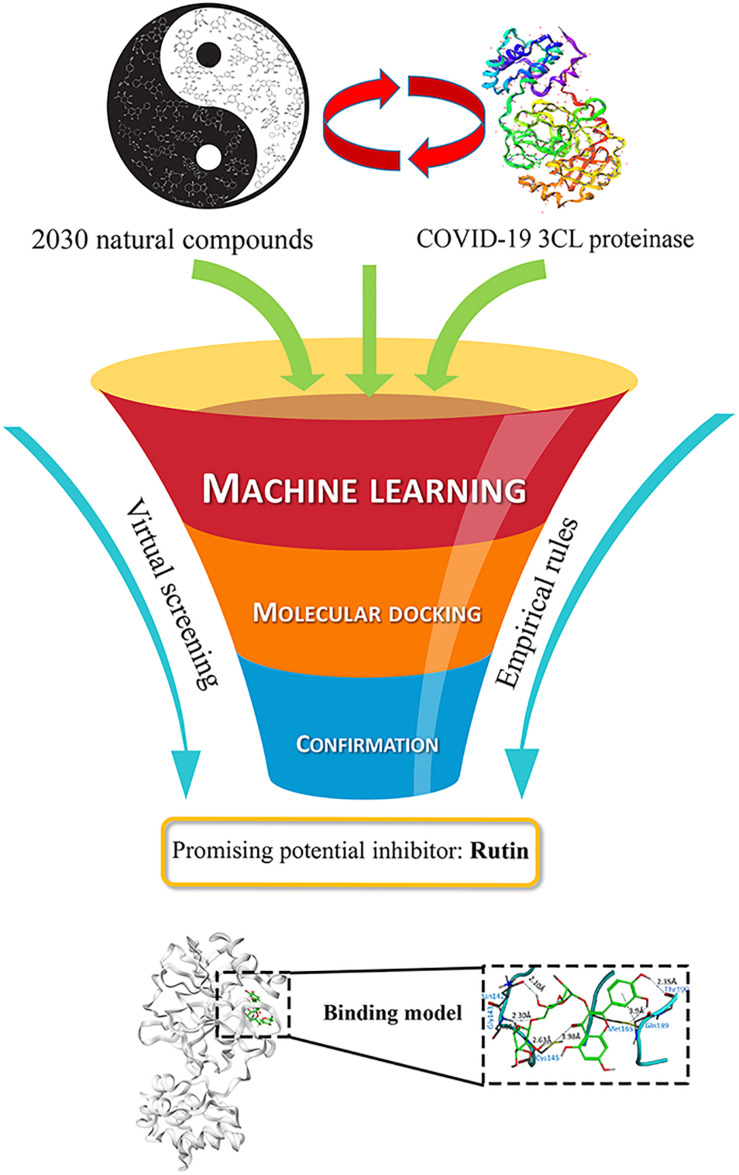
Schematic illustration of the anti-COVID-19 discover procedure of virtual screening.

Based on ML prediction and molecular docking procedures, six flavonoids were presumably considered as potential inhibitors of COVID-19 3CL proteinase. Among these inhibitors, the most likely one is the compound Rutin, depending on the comprehensive assessment. The summary of the results was shown in Figure 1, and the details were described and discussed as below.

### Performance of Machine Learning Classifiers

**FIGURE 2:**
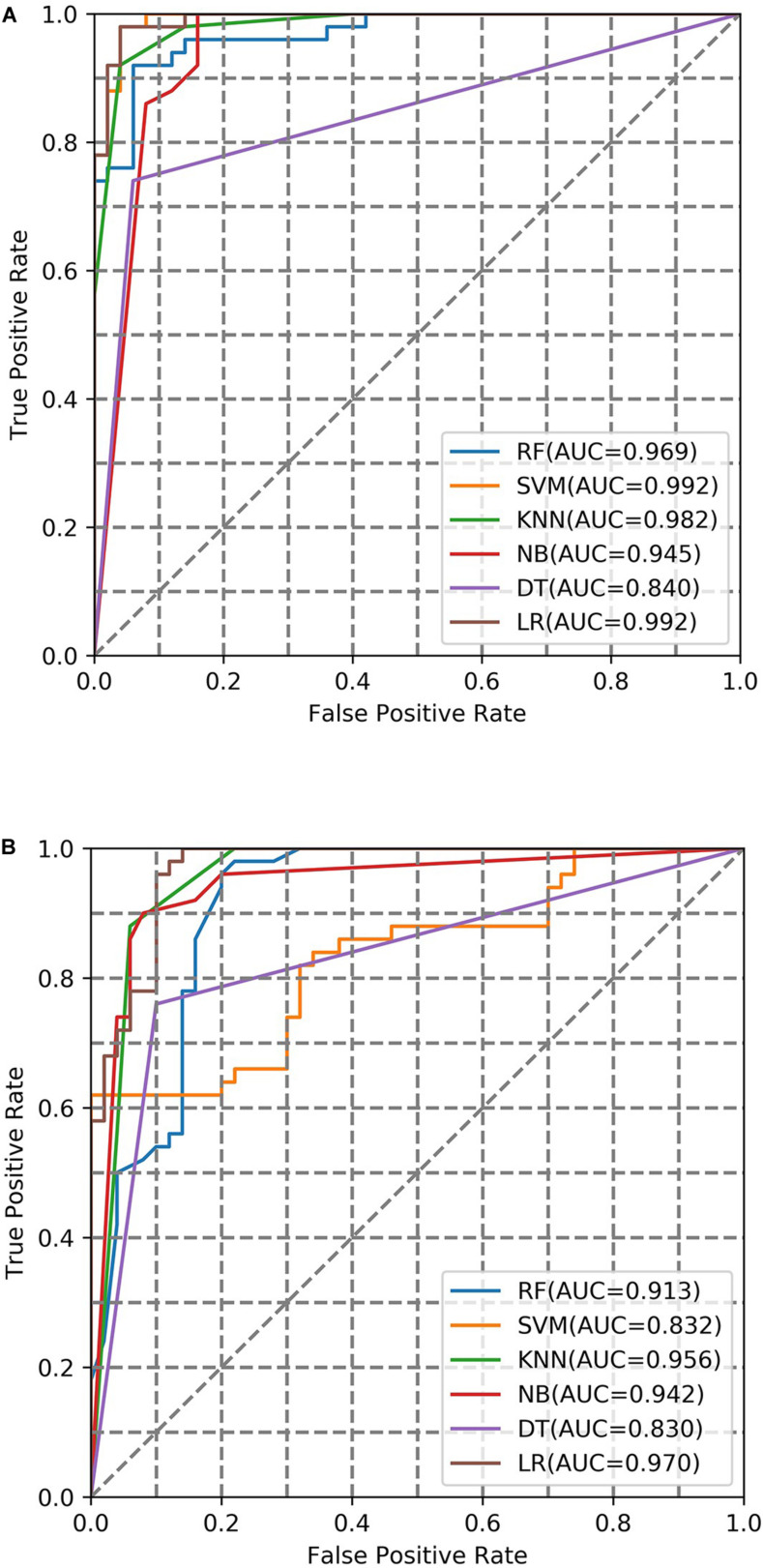
AUC curve of fivefold cross-validation with negative samples from inactive compounds in the case of different ML models. **(A,B)** are the AUC curve in the 1024-bit and 2048-bit length fingerprints, respectively.

To evaluate the performance of the training model, two factors were considered in a combinational way. These factors were (1) machine learning methods (LR, NB, DT, KNN, SVM, RF) and (2) fingerprint bits (128-bit, 256-bit, 526-bit, 1024-bit, 2048-bit). The AUC under different conditions were summarized (Figure 2 and Supplementary Table S1). On average, the performance of LR was best compared with the other five ML classifiers in the different fingerprint length. The AUC (0.976) of LR in the case of 2048-bit fingerprint was best compared with the case of other bits fingerprint. Finally, the Logistic Regression with 2048-bit fingerprint was selected to calculate the probability values (AUC) for the screening library.

### Analysis of the Original Ligand and Defining the Accuracy of Docking Protocol

In general, the molecular docking settings are often calibrated based on the experimental ligand-acceptor. Especially, the accuracy of docking protocol needs to be cross-checked by the training set used. However, it wasn’t long before the crystal structure of COVID-19 3CLpro complex analyzed and resolved. We found the exciting truth that the catalytic pocket of SARS 3CLpro (PDB: 3IWM) and COVID-19 3CLpro are exactly similar (Yang et al., 2006; Zhang et al., 2010; Supplementary Figure S1). This phenomenon highlights the key to confirm the binding mode of COVID-19 3CLpro complex, imitate the interaction between SARS 3CLpro and its approved inhibitors, and replicate the above binding mode to discover the potential COVID-19 3CLpro inhibitors.

The 6LU7 three-dimensional structure as well as the peptide inhibitor was analyzed. The inhibitor forms hydrogen bonds with different residues including His143, His164, Glu166, Gln189, and Thr190, and a weakly hydrogen binding with the residue Phe140. In addition, the possible formations of π-π stacking interaction with His41 and covalent bond with Cys145 were also observed. These observations further demonstrated that the original inhibitor would interact with key residues of COVID-19 3CLpro, in a similar way to that of the screening potential inhibitors against COVID-19. To confirm the proper docking protocol used, the original inhibitor was redocked five times, and the binding pose was in line with the previous crystal conformation.

### Secondary Screening With Molecular Docking

The Glide ligand docking module was used to conduct the initial screening based on the templates of SP and XP. As the screening dataset, 2030 natural compounds (Chinese Medicine compounds mostly) were added. We assumed that the predicted binding affinity reflected the real binding model. The screening results were ranked based on the docking score as an initial selection, and score section showed the clear properties between binding complexes and no-binding complexes (Wilsey et al., 2013). In other words, the lowest binding score means the best binding affinity. The results yielded 1340 compounds in the entry of Workspace. Further refinement of the above initial selected compounds were performed by analyzing and scoring with the MM-GBSA and the Pharmacophore Modeling.

The pharmacophore model was subsequently generated and optimized slightly. We clearly observed the main binding model between COVID-19 3CLpro and its original inhibitor. Especially, the generated model consisted of several features including one aromatic feature projecting to the residue Thr26, H-bond acceptors targeting Gly143, Cys145, and Glu166, and two H-bond donors targeting Glu166 and Thr190. Then, the optimized pharmacophore model was used to rescore the initial screened 1340 compounds. In this case, 98 hits were already reported to give reliability to our pharmacophore model. Since the catalytic pocket of COVID-19 3CLpro is extremely similar to SARS, the binding model is highly similar. The relevant factors of docking model should possibly follow the model of inhibitors binding with SARS-CoV 3CL protease (Chen et al., 2005; Jo et al., 2020). According to the comprehensive evaluation, the docking score and AUC of molecules were better than −7.0 kcal/mol and 0.70, respectively, which were considered more reliable. As mentioned above, the 32 hits were tentatively considered as the potential inhibitors.

### Visual Inspection

After visualizing the docked complexes carefully, we found that six agents with better docking scores compared to the others in the refinement list (Table 1 and Supplementary Table S2). Therefore, the six compounds were subsequently applied for further analysis. According to the binding sites of 6LU7 complex, the original inhibitor could form hydrogen bonds with surrounding residues in the active pocket. In addition, we noted that these compounds all presented electrostatic interaction with the residue Cys145, which is the key residue in the catalytic center of COVID-19 3CLpro complex. Besides, the residue Cys145 has also been approved to be the crucial residue forming within a radius of 6 Å around the catalytic center of SARS-CoV 3CLpro (Jacobs et al., 2013; Park et al., 2016). The key residues surrounding the active center of SARS-CoV 3CLpro include Hid41, Leu141, Gly143, Cys145, Glu166, and Asp187 (Li et al., 2016). Recently, the flavonoids have been approved to be the inhibitor targeting with the SARS-CoV 3CL protease (Jo et al., 2020), and they occupy the main pockets of the catalytic center including Asn 142, Glu166, and Gln189 residues. Interestingly, six screened compounds of our findings are also flavonoids. As mentioned above, both coronaviruses have the similar binding affinity, which means that the screened compounds could potentially occupy the catalytic active center of the protease very well, inhibiting the activity of protease to reduce the ability of virus copy.

**TABLE 1:** Six selected natural compounds according to the docked results and AUC.

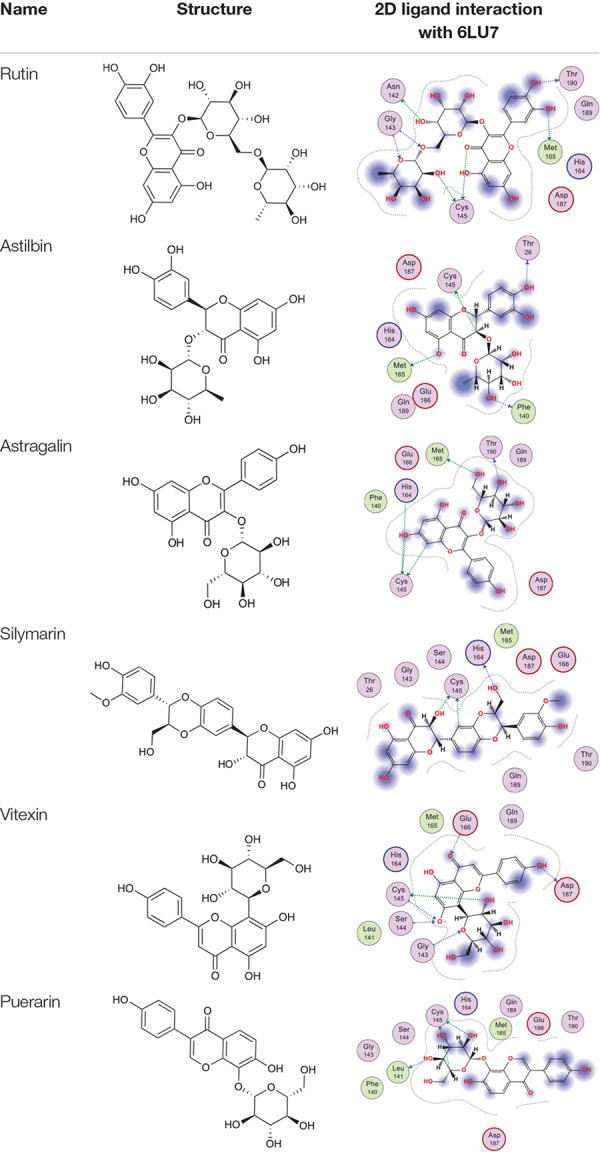

### Binding Modes of Rutin Against COVID-19 3CLpro

**FIGURE 3:**
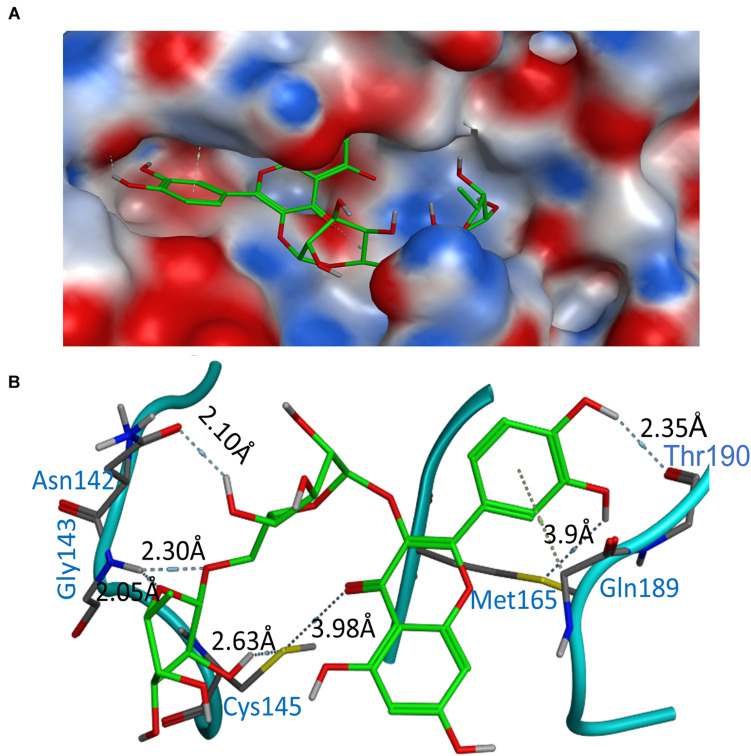
Binding modes of Rutin with 6LU7. **(A)** Electrostatic interaction between Rutin and crystal structure (6LU7) of COVID-19 3CLpro. **(B)** Interactions between Rutin and associated residues in the crystal structure (6LU7) of COVID-19 3CLpro. Blue and gray labels shown in the figure are residue names and interaction distance respectively.

According to the above analysis results, the compound Rutin (docking score: −9.16 kcal/mol and AUC: 0.990) was considered to be the most potential inhibitor compared with others. This compound was predicted to form the hydrogen bonds involving Cys145 (2.63Å), Asn142 (2.1 Å), Gly143 (2.3 Å), and Thr190 (2.35 Å), with additionally the possible formation of σ-π stacking interaction with Gln189 (Figures 3A,B). Notably, the major binding affinity was based on the presence of hydroxyl group, which presented the key in anchoring and blocking the substrate into the active pocket of catalytic center. Overall, the Rutin matched very well with 6LU7 binding pocket, indicating that it may be a potential inhibitor.

## Discussion

In summary, the rapid and efficient drug discovery procedure of virtual screening combined ML methods with molecule docking was performed. Based on the further evaluation and refinement, the most potential compound Rutin was highly screened, suggesting the compound might be active against the COVID-19 3CLpro. Moreover, two flavonoids, baicalin and baicalein, have recently been identified as the novel, natural product inhibitors of 3CL protease *in vitro* (Su et al., 2020), and the flavonoids could be potential anti-COVID-19 inhibitors (Liu et al., 2020). In addition, the Rutin has been proved to be against the flu viruses, and Rutin tablets have been used in clinic for many years in China. Therefore, Rutin may be a potential inhibitor against COVID-19 3CLpro. There are also some limits of our approach. The number of compounds used in the machine learning procedure is not enough and we still contribute to this database. In addition, we are still keeping an eye on the latest development of COVID-19 researches. We will collect more related compounds and update our machine learning training data. Simultaneously, there is still room for progress in machine learning procedure, and the deep machine learning used in the drug screening would be perfect for filling the gap. This article is just the beginning of the research on the combination of machine learning and molecular docking. We believe that the combination of molecular docking and machine learning will bring more surprises for the discovery of drugs. *In vitro* and *in vivo* studies will be designed and carried out to validate the therapeutic effects of Rutin for COVID-19 in the future (Chen et al., 2020).

## Data Availability Statement

All datasets generated for this study are included in the article/Supplementary Material.

## Author Contributions

ZX and LY were responsible for the molecular docking, machine learning, and data analysis. XZ, QZ, ZY, YL, and SW devoted themselves to the compounds data collection. WL committed himself to the manuscript to revise and polish. All authors contributed to the article and approved the submitted version.

## Conflict of Interest

The authors declare that the research was conducted in the absence of any commercial or financial relationships that could be construed as a potential conflict of interest.

## Supplementary Material

FIGURE S1 The superpose structure of COVID-19 3CLpro (gray) and SARS-3CLpro (green). The catalytic dyad (His41 and Cys145) are shown as sticks.

TABLE S1 The AUC score of fivefold cross-validation.

TABLE S2 Analyzed results of six selected compounds.
