# Supplementary material for: Discovery of Potential Flavonoid Inhibitors Against COVID-19 3CL Proteinase Based on Virtual Screening Strategy
Source: Front Mol Biosci. 2020 Sep 29;7:556481. doi: 10.3389/fmolb.2020.556481 (PMC7561382; doi:10.3389/fmolb.2020.556481)
Supplement: FIGURE S1 — The superpose structure of COVID-19 3CLpro (gray) and SARS-3CLpro (green). The catalytic dyad (His41 and Cys145) are shown as sticks. [file Table_1.DOC]

**Supplementary materials:**


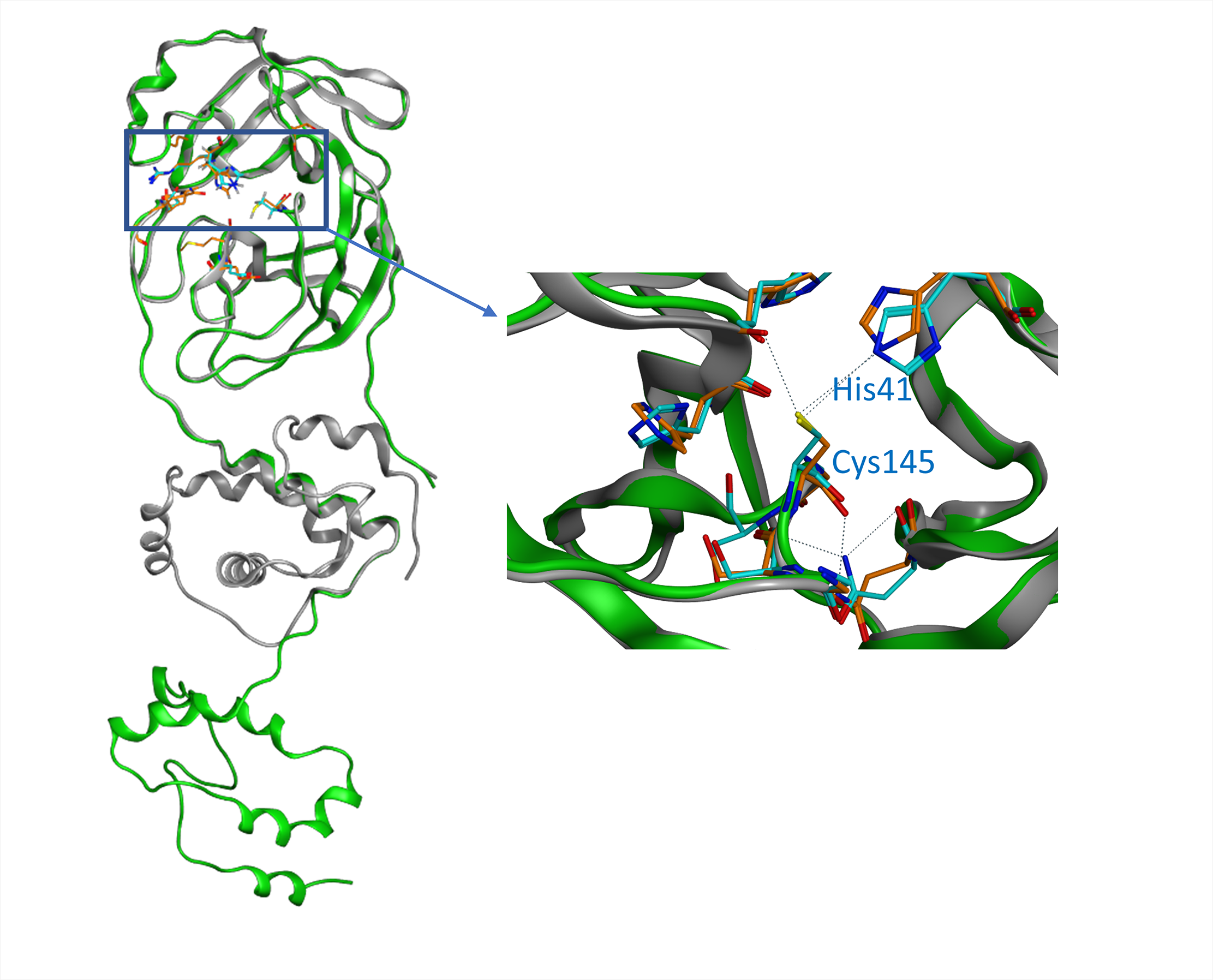


**Figure S1** The superpose structure of COVID-19 3CLpro (gray) and SARS-3CLpro (green). The catalytic dyad (His41 and Cys145) are shown as sticks.

Table S1. The AUC score of five-fold cross-validation.

| **Classifier** | **Fingerprint** | **Test 1** | **Test 2** | **Test 3** | **Mean ± SD** |
| --- | --- | --- | --- | --- | --- |
| SVM | 128-bit | 0.904 | 0.958 | 0.926 | 0.929±0.027 |
|  | 256-bit | 0.500 | 0.984 | 0.500 | 0.661±0.279 |
|  | 512-bit | 0.839 | 0.989 | 0.973 | 0.934±0.082 |
|  | 1024-bit | 0.960 | 0.992 | 0.975 | 0.976±0.016 |
|  | 2048-bit | 0.957 | 0.993 | 0.832 | 0.928±0.084 |
| RF | 128-bit | 0.900 | 0.945 | 0.935 | 0.926±0.023 |
|  | 256-bit | 0.910 | 0.977 | 0.924 | 0.937±0.035 |
|  | 512-bit | 0.954 | 0.986 | 0.973 | 0.971±0.016 |
|  | 1024-bit | 0.915 | 0.969 | 0.968 | 0.951±0.031 |
|  | 2048-bit | 0.930 | 0.978 | 0.913 | 0.941±0.034 |
| NB | 128-bit | 0.766 | 0.807 | 0.796 | 0.789±0.021 |
|  | 256-bit | 0.740 | 0.700 | 0.730 | 0.723±0.021 |
|  | 512-bit | 0.710 | 0.790 | 0.770 | 0.757±0.042 |
|  | 1024-bit | 0.780 | 0.840 | 0.830 | 0.817±0.032 |
|  | 2048-bit | 0.800 | 0.850 | 0.830 | 0.827±0.025 |
| LR | 128-bit | 0.868 | 0.954 | 0.894 | 0.905±0.044 |
|  | 256-bit | 0.936 | 0.983 | 0.947 | 0.955±0.025 |
|  | 512-bit | 0.956 | 0.989 | 0.964 | 0.969±0.017 |
|  | 1024-bit | 0.956 | 0.992 | 0.972 | 0.973±0.018 |
|  | 2048-bit | 0.967 | 0.993 | 0.970 | **0.976±0.014** |
| KNN | 128-bit | 0.897 | 0.933 | 0.917 | 0.916±0.018 |
|  | 256-bit | 0.918 | 0.940 | 0.932 | 0.930±0.011 |
|  | 512-bit | 0.936 | 0.966 | 0.959 | 0.954±0.016 |
|  | 1024-bit | 0.945 | 0.982 | 0.956 | 0.961±0.019 |
|  | 2048-bit | 0.925 | 0.935 | 0.956 | 0.939±0.016 |
| DT | 128-bit | 0.867 | 0.853 | 0.871 | 0.864±0.010 |
|  | 256-bit | 0.904 | 0.916 | 0.911 | 0.910±0.006 |
|  | 512-bit | 0.898 | 0.927 | 0.905 | 0.910±0.015 |
|  | 1024-bit | 0.934 | 0.945 | 0.913 | 0.931±0.017 |
|  | 2048-bit | 0.903 | 0.956 | 0.942 | 0.933±0.027 |

**Table S2** Analyzed results of six selected compounds.

| Name | CAS | Score | E_strain | MM-GBSA | Weight | logP | Acceptor | Donor |
| --- | --- | --- | --- | --- | --- | --- | --- | --- |
|  |  | (kcal/mol) | (kcal/mol) | (kcal/mol) |  |  |  |  |
| Rutin hydrate | 207671-50-9 | -9.16 | 32.21 | -65.61 | 610.52 | -1.11 | 16 | 10 |
| Astilbin | 29838-67-3 | -8.22 | 13.73 | -55.63 | 449.39 | 0.69 | 11 | 6 |
| Silymarin | 65666-07-1 | -8.01 | 10.45 | -66.12 | 481.43 | 2.76 | 10 | 4 |
| Astragalin | 480-10-4 | -7.93 | 15.71 | -53.21 | 448.38 | 0.04 | 11 | 7 |
| Vitexin | 3681-93-4 | -7.83 | 63.59 | -46.86 | 431.37 | 0.18 | 10 | 6 |
| Puerarin | 3681-99-0 | -7.68 | 14.38 | -46.86 | 432.38 | -0.43 | 10 | 6 |

**Note,** Score is the binding energy between selected compounds and target. Weight is molecular weight; E strain is the deformational energy before and after the molecule matching to the pocket of target. MM-GBSA presents the molecular mechanics with generalized Born and surface area. Acceptor is hydrogen bond receptor. Donor is hydrogen bond donor. LogP is the oil-water partition coefficient. P-value is the probability of the prediction by LR model calculation.
